# Advanced Lab-on-Fiber Optrodes Assisted by Oriented Antibody Immobilization Strategy

**DOI:** 10.3390/bios12111040

**Published:** 2022-11-17

**Authors:** Sarassunta Ucci, Sara Spaziani, Giuseppe Quero, Patrizio Vaiano, Maria Principe, Alberto Micco, Annamaria Sandomenico, Menotti Ruvo, Marco Consales, Andrea Cusano

**Affiliations:** 1 Institute of Biostructures and Bioimaging, National Research Council of Italy, Via P. Castellino, 111, 80131 Naples, Italy; 2 Optoelectronics Group, Engineering Department, University of Sannio, c.so Garibaldi 107, 82100 Benevento, Italy; 3 Centro Regionale Information Communication Technology (CeRICT Scrl), 82100 Benevento, Italy

**Keywords:** oriented antibody, cancer biomarker, biosensing, surface plasmon resonance, optical fiber biosensor, lab-on-fiber technology

## Abstract

Lab-on-fiber (LoF) optrodes offer several advantages over conventional techniques for point-of-care platforms aimed at real-time and label-free detection of clinically relevant biomarkers. Moreover, the easy integration of LoF platforms in medical needles, catheters, and nano endoscopes offer unique potentials for in vivo biopsies and tumor microenvironment assessment. The main barrier to translating the vision close to reality is the need to further lower the final limit of detection of developed optrodes. For immune-biosensing purposes, the assay sensitivity significantly relies on the capability to correctly immobilize the capture antibody in terms of uniform coverage and correct orientation of the bioreceptor, especially when very low detection limits are requested as in the case of cancer diagnostics. Here, we investigated the possibility to improve the immobilization strategies through the use of hinge carbohydrates by involving homemade antibodies that demonstrated a significantly improved recognition of the antigen with ultra-low detection limits. In order to create an effective pipeline for the improvement of biofunctionalization protocols to be used in connection with LoF platforms, we first optimized the protocol using a microfluidic surface plasmon resonance (mSPR) device and then transferred the optimized strategy onto LoF platforms selected for the final validation. Here, we selected two different LoF platforms: a biolayer interferometry (BLI)-based device (commercially available) and a homemade advanced LoF biosensor based on optical fiber meta-tips (OFMTs). As a clinically relevant scenario, here we focused our attention on a promising serological biomarker, Cripto-1, for its ability to promote tumorigenesis in breast and liver cancer. Currently, Cripto-1 detection relies on laborious and time-consuming immunoassays. The reported results demonstrated that the proposed approach based on oriented antibody immobilization was able to significantly improve Cripto-1 detection with a 10-fold enhancement versus the random approach. More interestingly, by using the oriented antibody immobilization strategy, the OFMTs-based platform was able to reveal Cripto-1 at a concentration of 0.05 nM, exhibiting detection capabilities much higher (by a factor of 250) than those provided by the commercial LoF platform based on BLI and similar to the ones shown by the commercial and well-established bench-top mSPR Biacore 8K system. Therefore, our work opened new avenues into the development of high-sensitivity LoF biosensors for the detection of clinically relevant biomarkers in the sub-ng/mL range.

## 1. Introduction

In recent years, the need for innovative and ever-sensitive methods of detecting chemical and biological analytes has grown considerably. The interest in developing new dedicated biosensors has expanded in parallel, both in academia and industry, while attempting to exploit their potentiality in various new fields of application [1,2,3]. Among all biosensors, optical ones represent the most promising types and in most cases have been developed to exploit the ability of surface-immobilized antibodies to recognize the target analytes with high affinity and specificity [4,5].

In such optical immune-biosensors, antibody immobilization is a crucial step because it should ideally maintain the antigen recognition ability as in solution [6]. Immobilization on a solid surface can be achieved in a random or site-oriented manner [7], but generally, only in the second instance is the antibody properly positioned to allow the most productive interaction of the fragment antigen-binding (Fab) with the analyte [8,9], thus optimizing the biosensor’s performances [10]. Among the various possible methods, site-oriented antibody immobilization can be realized via the affinity capture of the fragment crystallizable (Fc) region [8,11,12]; or by modulating the buffer pH, the immobilization density, the antibody-surface charge distribution, or the antibody hydrophilicity [13,14,15]; or, again, via a chemical modification of the oligosaccharides present in the hinge region [8,16,17,18].

Although much progress has been made in developing optical sensors in small and easy-to-use systems, many applications such as point-of-care (POC) [19,20] diagnostics and in vivo biosensing still require a miniaturized probe that is able to perform measurements at precise locations that are often hard to reach with common lab-on-chip approaches [21,22]. The intrinsic property of optical fibers (OFs) to conduct light to a remote location makes them an ideal platform to meet this demand. The motivation to combine the good performance of photonic biosensors on a chip with the unique advantages of OFs has led to the development of the so-called lab-on-fiber (LoF) technology [23]. Through the integration of suitable biomaterials defined at the micro- and nanoscale, an OF can be engineered to fulfill a number of tasks: to become a biosensor [24,25,26,27,28], a high-resolution molecular-recognition tool [29,30], or a probe for drug delivery [31,32,33]. Moreover, the biocompatible nature of optical fibers combined with their simple integration in medical needles, catheters, and minimally invasive clinical tools would allow the immunoassay to be performed directly in vivo (inside the patient’s body).

A great breakthrough in the LoF roadmap has been the integration of plasmonic metasurfaces on an OF tip [25,34,35], which led to the new paradigm of the OF “meta-tips” (MTs). After a first proof-of-concept dedicated to the application of OFMTs for beam steering [35], the manifold degrees of freedom for optimizing the surface sensitivity of OFMTs to local variations of the refractive index were recently exploited to demonstrate the capability of detecting biomolecular interactions using the well-known biotin–streptavidin pair with an ultra-low limit of detection (LOD) [25].

**Figure 1:**
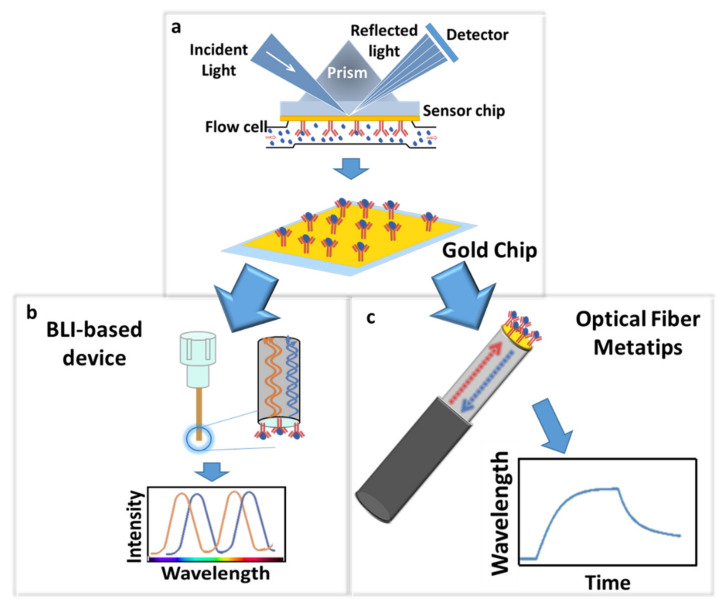
Schematic representation of the exploited approach: biofunctionalization protocol optimization using a gold chip (GC)-based mSPR device (**a**) and its successive transfer to a BLI-based platform that exploited a plastic optical fiber (ARG2 biosensor) (**b**) and a LoF platform based on a highly sensitive OFMT (**c**) for Cripto-1 detection evaluation.

The reported evidence demonstrates that LoF optrodes offer several advantages over conventional techniques for POC platforms aimed at real-time and label-free detection of clinically relevant biomarkers. Moreover, the easy integration of LoF platforms in medical needles, catheters, and nano endoscopes offers unique potentials for in vivo biopsies and tumor microenvironment assessment. The main barrier to translate the vision close to the reality is the need to further lower the final limit of detection of developed optrodes. The sensitivity and specificity of biosensors rely significantly on the ability to properly immobilize the capture antibody in terms of the uniform coverage and correct orientation of the bioreceptor, especially for the very low concentration detection limits required for cancer diagnostics. Here, we investigated the possibility of improving the immobilization strategies through the use of hinge carbohydrate oxidation to provide a target for site-directed immobilization. Our strategy involved homemade antibodies and demonstrated a significantly improved recognition of the antigen with ultra-low detection limits. To create an effective pipeline for the improvement of biofunctionalization protocols to be used in connection with LoF platforms, we tested and assessed the protocol with a commercial mSPR system using a gold chip (GC)-based device [36] and successively transferred it to two different LoF platforms: (i) a BLI-based platform that exploited a plastic optical fiber [37] and (ii) an advanced LoF biosensor based on highly sensitive OFMTs (Figure 1).

As a case study, we focused our attention on Cripto-1, a promising serological biomarker and the original member of the EGF-CFC/FRL1/Cryptic family of proteins. In both the soluble and cell-associated forms, it is involved in embryonic development and, in adults, in carcinogenesis [38,39]. Soluble Cripto-1 is indeed overexpressed in various cancers [40,41,42,43,44,45,46] in which it regulates cell proliferation [47], survival [48], migration [49], invasion [50], and tumor angiogenesis [51,52], making it an interesting biomarker for several diseases. The main methods used to study and identify Cripto-1 are a sandwich-type enzyme-linked immunosorbent assay (ELISA) [40] and Western blotting [53,54], which are both relatively expensive and time-consuming. So far, no biosensors capable of detecting Cripto-1 in a label-free manner have been described; our study, therefore, had the further aim of exploring the optimal conditions for its quantification using cheaper and faster detection systems.

The reported experiments suggested the huge potentiality of the novel OFMT-based biosensing platform to perform Cripto-1 detection, thereby opening new avenues into the development of high-sensitivity LoF biosensors for cancer diagnosis.

## 2. Materials and Methods

### 2.1. Instrumentation and Reagents

The SPR experiments were performed with the Biacore 8K system (Cytiva, Marlborough, MA, USA). The gold chip biosensor (SIA Au), HBS-EP+ running buffer (final composition: 0.01 M HEPES, 0.15 M NaCl, 0.003 M EDTA, and 0.05% *v*/*v* Surfactant P20), N-hydroxy succinimide (NHS) 1-ethyl-3-(3-dimethylamino-propyl) carbodiimide (EDC), 1 M ethanolamine-HCl pH 8.5 (MEA) and 5 mM NaOH were all from Cytiva (Marlborough, MA, USA). The CT(PEG)12 carboxy-PEG-thiol (SH-PEG-COOH), QuantaRed Enhanced Chemifluorescent HRP Substrate, sodium cyanoborohydride (NaBH_3_CN), Dulbecco’s Modified Eagle’s Medium (DMEM), and NanoDrop Onec Microvolume UV–vis spectrophotometer were obtained from Thermo Fisher Scientific (Waltham, MA, USA). The phosphate-buffered saline (PBS), SH-PEG-NH_2_, ethylene glycol, sodium periodate, sodium acetate, and bovine serum albumin (BSA) were obtained from Sigma Aldrich (St. Louis, MO, USA). The peroxidase (HRP) AffiniPure goat anti-mouse IgG was obtained from Jackson ImmunoResearch (Cambridge, UK, code n° 115035003). The mouse anti-human alpha fetoprotein that was used as an unrelated antibody was obtained from Bio-Rad (code n° MCA5862G). The Lucifer Yellow CH (LyCH) dilithium salt was obtained from MP Biomedical (Solon, OH, USA). The Amicon^®^ Ultra 0.5 mL 10K centrifugal filters were obtained from Merck (Burlington, MA, USA). The biolayer interferometry instrument (BLItz) and the amine reactive (AR2G) sensors were obtained from the Sartorius Company (Gottingen, Germany). The EnSight multimode plate reader was obtained from PerkinElmer (Waltham, MA, USA). The realization of the pattern of nanoholes was carried out using a focused ion beam (FIB) milling system (Quanta 200 3D Dual-Beam System from the FEI Company, Eindhoven, The Netherlands) integrated with a scanning electron microscope (SEM).

### 2.2. Biofunctionalization Protocol

#### 2.2.1. Oxidized 1B4 Characterization

The murine monoclonal 1B4 anti-Cripto-1 antibody (1B4) was generated and characterized as reported in Focà et al. [55]. The 1B4 was incubated with 20 mM sodium periodate (NaIO_4_) (1:1) in 0.1 M sodium acetate (NaAc) at pH 5.0 for 60 min with shaking in the dark at room temperature (RT). The oxidation reaction was stopped by adding 10% ethylene glycol for 10 min. Finally, the excess NaIO_4_ was removed using Amicon Ultra centrifugal filters that were pre-equilibrated with 10 mM PBS. The amount of 1B4 was determined using a UV–vis spectrophotometer (NanoDrop Onec). The oxidized 1B4 (Ox-1B4) was used immediately or stored at 4 °C until use (generally no longer than one month). The effective number of aldehyde groups available on the Ox-1B4 was determined by labeling with LyCH, a hydrazide-containing dye. For this analysis, the same amounts of Ox-1B4 and unmodified 1B4 (Un-1B4) were incubated with LyCH (at an antibody-to-dye ratio of 1:250) for 1 h in the dark at RT in 10 mM NaAc at pH 5.0. Then, the solution was filtered with Amicon Ultra centrifugal filters that were pre-equilibrated in 10 mM NaAc at pH 5 at 10,000× *g* for 10 min at 4 °C. Then, 0.1 M NaBH_3_CN in 10 mM NaAc pH 5.0 was added to the mixture and allowed to react for 30 min. The excess LyCH and NaBH_3_CN were removed via ultrafiltration in 10 mM PBS. The samples of labeled antibodies and the relative standard curve were read at 430 nm with the EnSight multimode plate reader from PerkinElmer.

A direct ELISA was conducted to assess the Ox-1B4’s recognition efficiency. Different concentrations of Cripto-1 (0–1.5–6–24 nM) in 10 mM PBS were absorbed in a 96-well polystyrene microplate overnight at 4 °C. After 3% *w*/*v* BSA passivation for 1 h, the Ox-1B4 and Un-1B4 (1 μg/mL in PBS buffer) were added (100 µL) and incubated for 1 h. Then, a species-specific HRP-conjugated anti-mouse antibody was used to detect and quantify the bound antibodies. Following multiple washes, the wells were finally treated with QuantaRed Enhanced Chemifluorescent HRP Substrate and the fluorescence was read using the EnSight multimode plate reader set at 570 nm for excitation and 590 nm for emission.

#### 2.2.2. Immobilization on a Flat Gold Surface

In order to verify the biofunctionalization protocol, the 1B4 antibody was anchored in oriented and random fashions on a planar gold surface. For this purpose, a microscope glass slide coated with two thin layers of titanium (Ti) (~3 nm) and gold (Au) (~30 nm) was cleaned for 1 min with a piranha solution (a combination of sulfuric acid (H_2_SO_4_) and hydrogen peroxide (H_2_O_2_)) at a ratio of 3:1. After cleaning, the piece was assembled in a ProPlate^®^ Grace Bio-Labs microarray system (a microplate cassette that allows the obtaining of 24 separate wells when the experiment reactions are carried out) (Grace Bio-Labs, Bend, OR, USA). The wells were incubated with a solution of the thiols SH-PEG-COOH or SH-PEG-NH_2_ bearing free carboxyl or amino groups at 1 mM in 98% ethanol (EtOH) for 16 h at 4 °C to form an ordered self-assembled monolayer (SAM) [56,57]. The SH-PEG-COOH (47.8 Å) and SH-PEG-NH_2_ thiols were characterized by a short linker and (126 Å), allowing us to reduce the distance between the antibody and the gold surface. The presence of PEG was also beneficial for the inherent antifouling properties of this material. The Ox-1B4 was incubated at different concentrations (0–5–15–30 μg/mL in 10 mM NaAc at pH 5.0) directly on the SH-PEG-NH_2_ for 1 h at RT to form the corresponding imine bonds with the free NH_2_ group on the surface and then reduced with 0.1 M NaBH_3_CN in 10 mM NaAc at pH 5.0. The Un-1B4 coupling was performed on the SH-PEG-COOH following preactivation with a mixture composed of 0.8 M EDC and 0.2 M NHS in 10 mM NaAc at pH 5.0 for 15 min. The Un-1B4 at different concentrations (0–5–15 μg/mL) in 10 mM NaAc at pH 5.0 was then incubated for 1 h at RT. After 3% *w*/*v* BSA passivation for 1 h, an anti-mouse HRP-conjugated secondary antibody was used to detect and quantify the bound antibody. QuantaRed Enhanced Chemifluorescent HRP Substrate was used for the fluorescent detection of the peroxidase activity as described earlier.

#### 2.2.3. SPR Assay: Analyses on Gold Chip (GC) Biosensor

GC biosensors offer a flat gold surface mounted on a support for the creation of customized surfaces. The GC biosensor was treated with the piranha solution for 1 min and incubated with an SH-PEG-COOH solution in 98% EtOH for 16 h at 4 °C. The GC biosensor was then washed with fresh EtOH to remove the unbound molecules, dried with nitrogen, and placed in the Biacore 8K. The Ox-1B4 was immobilized on the active flow cell 2 (FC2) of four of the eight channels using the aldehyde coupling chemistry with the following steps: activation with 0.4 M EDC/0.1 M NHS (1:1, *v*/*v*) in H_2_O for 180 s at a flow rate of 10 μL/min; 5 mM hydrazine in H_2_O (420 s at 10 μL/min); 1 M ethanolamine-HCl at pH 8.5 (MEA) for 420 s at 10 μL/min; Ox-1B4 in 10 mM NaAc at pH 5.0 (420 s at 10 μL/min); and 0.1 M NaBH_3_CN in 0.1 M NaAc at pH 4.0 with a contact time of 1200 s at 2 μL/min. The Un-1B4 was randomly immobilized using the amine coupling chemistry on the FC2 of the other four channels. The flat surface was activated by injecting a 1:1 (*v*/*v*) mixture of 0.4 M EDC and 0.1 M NHS in water for 420 s. The Un-1B4 immobilization was carried out in 10 mM NaAc at pH 5.0 at a concentration of 30 μg/mL with a contact time of 420 s followed by the COOH deactivation with 1 M ethanolamine-HCl pH 8.5 (MEA) for 420 s at 10 μL/min. The unrelated antibody in the oriented and random configurations was immobilized on the related FC1. After antibody immobilization, the unfunctionalized gold surface was blocked by exposing the chip to 1 mg/mL BSA in HBS-EP+ for 900 s at a flow rate of 5 μL/min. The Cripto-1 detection was performed using an independent injection of samples at different concentrations (0.05–0.5–2.5–5–12.5 nM) in HBS-EP+ and DMEM (which contained peptides, vitamins, and amino acids and was completed with 1% pen/strep and 10% FBS) for 120 s at 30 μL/min.

The data were processed and analyzed using Biacore 8K Evaluation Software Version 3.0 (Cytiva, Marlborough, MA, USA). The responses recorded on the FC1 were subtracted from those in the corresponding FC2. The responses from the nearest buffer blank injection were subtracted from the reference subtracted data (FC2-FC1) to yield double-referenced data. Statistical analyses were performed using a one-tailed paired t-test (95% confidence interval) to assess the statistical significance in recognition of the same Cripto-1 concentrations using the oriented 1B4 rather than the random one.

### 2.3. Lab-on-Fiber Sensing Platforms

#### 2.3.1. Bio-Layer Interferometry Analyses

The measurements were performed on a single-channel BLItz system using AR2G biosensors that bore a surface with a high density of carboxylic acids with a low propensity for non-specific interactions. All samples were exposed to the sensor tips in the drop mode (4 μL), while the running buffer (PBS) was used in tubes at 200 μL according to manufacturer’s instructions.

The Ox-1B4 was immobilized on the AR2G biosensors using the aldehyde coupling chemistry with the following steps: activation with 0.4 M EDC/0.1 M NHS (1:1, *v*/*v*) in H_2_O for 120 s; 5 mM hydrazine in H_2_O (180 s); 1 M ethanolamine-HCl at pH 8.5 (MEA) for 60 s; Ox-1B4 in 10 mM NaAc at pH 5.0 (180 s); and 0.1 M NaBH_3_CN in 0.1M NaAc at pH 4.0 with a contact time of 180 s. Un-1B4 was randomly immobilized using the amine coupling chemistry. The AR2G biosensor surface was activated by a 1:1 (*v*/*v*) mixture of 0.4 M EDC and 0.1 M NHS in water for 120 s. The Un-1B4 immobilization was carried out in 10 mM NaAc at pH 5.0 at a concentration of 30 μg/mL with a contact time of 180 s. The exceeding reactive esters were blocked with NaOH 0.1M for 30 s and MEA for 60 s.

After the immobilization of the antibodies, the AR2G biosensors were incubated with Cripto-1 solutions at different concentrations (0–12.5–50–500 nM). All data were processed using min–max normalization [58] and statistical analyses were performed using a one-tailed paired *t*-test to assess the statistical significance in recognition of the same Cripto-1 concentrations using the oriented 1B4 rather than the random one.

#### 2.3.2. Optical Fiber Meta-Tips (OFMTs)

**Figure 2:**
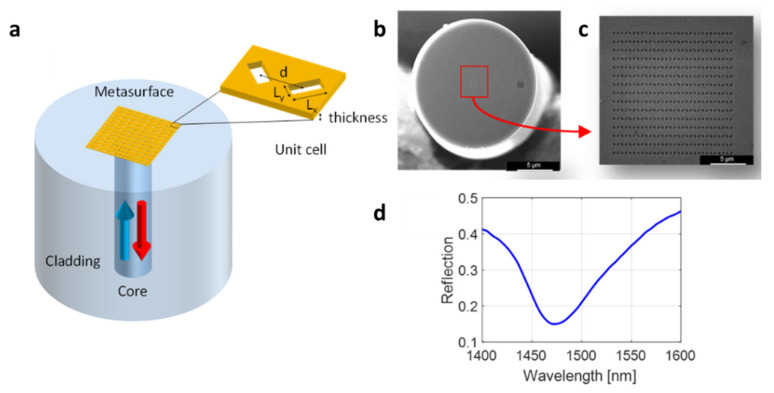
Basic schematic of an OFMT (**a**); SEM image of an OFMT (**b**); zoomed image of the nanopattern of a phase-gradient OFMT (**c**); reflection spectrum of a realized phase-gradient MT probe (**d**).

The metasurface-assisted LoF biosensing platform used in this work was based on phase-gradient plasmonic OFMTs [25]. They consisted of 2D arrays of rectangular apertures with a spatially modulated size in a thin gold layer deposited onto the tip of an OF (Figure 2a). The nanoantennas were arranged in order to create a phase gradient, which guaranteed a greater confinement of the electric field compared with the gradient-free counterpart, thus resulting in a higher surface sensitivity [25]. The realization of an OFMT requires three main steps: (i) metallic layer deposition, (ii) gold layer patterning, and (iii) gold layer confinement. In the first step, the cleaved end of a standard single-mode OF (Corning SMF-28) was coated with two thin layers of Ti (~3 nm) and Au (~30 nm) via an electron beam evaporation system. The pattern was realized by using the FEI Quanta 200 3D focused ion beam milling system within a ~14 × 14 μm^2^ area centered around the fiber core. Figure 2b,c show the SEM image of a realized metasurface featuring an array of rectangular holes with a size (L_x_ × L_y_) of 300 nm × 120 nm and an orientation of ±45° and spaced with a period d = 500 nm. Finally, the metallic layer was ablated via a UV laser while leaving a 50 × 50 μm^2^ gold square around the central part of the fiber end facet to reduce the gold area not affected by the surface wave generated by the OFMT.

The optical measurements were carried out using an optoelectronic setup that essentially consisted of a polarized broadband light source, an optical spectrum analyzer, a 1 × 2 optical coupler, and a polarization controller for the optimization of the OFMT resonant dip [25]. With the aim of performing the binding experiment in the buffer liquid solutions, the pattern within the metallic film was designed and realized to excite a plasmonic resonant dip in the reflectivity spectrum centered at around 1480 nm for a surrounding refractive index of 1.333 (Figure 2d).

#### 2.3.3. OFMT Biofunctionalization

The optimized biofunctionalization protocol was transferred to the OFMTs to perform the detection of the Cripto-1 biomarker. In particular, the OF tip was first cleaned via the piranha solution and incubated overnight with 1 mM SH-PEG-NH_2_ thiols in EtOH at 4 °C. After that, the probe was immersed for a few minutes in EtOH and then subjected to a rinsing phase in 10 mM NaAc at pH 5.0 until reaching spectral stability. The next step was the incubation with 15 µg/mL Ox-1B4 in NaAc. After washing, the probe was dipped in NaBH_3_CN to stabilize the covalent bond and then washed again in NaAc. The probe was immersed in PBS to restore a physiological pH and finally treated with 3% *w*/*v* BSA in PBS to passivate the sensor surface followed by a rinse in PBS. Then, the OFTM probe was exposed to different concentrations of Cripto-1 (0.05–0.5–5 nM).

## 3. Results and Discussion

To create an effective pipeline for the improvement of biofunctionalization protocols, we first optimized the protocol using the mSPR device, and then we transferred the optimized strategy to the LoF platforms selected for the final validation: a BLI-based device (commercially available) and an advanced homemade LoF biosensor based on OFMTs.

### 3.1. Biofunctionalization Protocol

#### 3.1.1. Characterization of Ox-1B4

**Figure 3:**
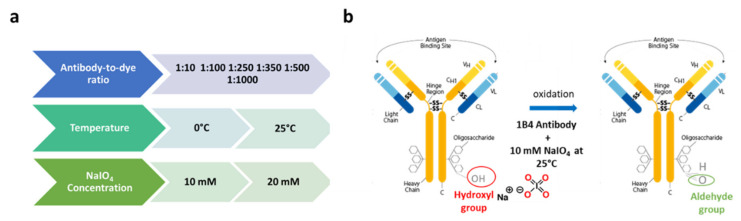
Optimization parameters: (i) antibody-to-dye ratio for the labeling, (ii) temperature, and (iii) NaIO_4_ for the oxidation reaction (**a**). Graphical representation of the optimized protocol (**b**).

Here, we investigated how the orientation of an antibody immobilized on the surface of an optical biosensor played a fundamental role in the determination of the overall performances of label-free biosensors toward the target analyte. We thus comparatively evaluated the detection ability of commercial as well as prototypical optical biosensing platforms when the capturing antibody was attached in a random or oriented manner. Cripto-1 detection with a high affinity (KD = 0.24 nM) and a highly specific 1B4 monoclonal antibody [55] was chosen as a case study given the current lack of cheap and fast assays for detecting this oncogene with biosensing platforms. For the oriented immobilization of 1B4, we optimized a protocol based on the NaIO_4_ oxidation of hinge carbohydrates to form reactive aldehydes that were subsequently captured by amines and hydrazides present on the surface, forming stable covalent bonds. Antibody immobilization through carbohydrates is in principle the ideal solution to optimize antigen recognition, since anchorage through the hinge region, which is long, flexible, and far from the complementarity-determining regions (CDRs), exposes them on the surface of the sensor almost as if they were bound to Fc receptors [7]. The 1B4 antibody is an IgG2b subtype [59], while the Fc fragment in its native state is glycosylated at the conserved Asn297 site with two-branched oligosaccharide chains [60]; therefore, many aldehyde groups are generated following periodate oxidation. Since even a single aldehyde is sufficient for the immobilization, the mild conditions chosen (10 mM NaIO_4_ at RT) might have been appropriate for generating a sufficient number of anchoring groups [61]. The effective number of aldehyde groups available for coupling was experimentally determined using a LyCH labeling operation, as reported elsewhere [61]. A step in the optimization suggested that the best conjugation between LyCH and Ox-1B4 occurred using a 1:250 ratio between the antibody and the dye (Figure S1). We also optimized the reaction conditions to obtain the highest possible number of aldehyde groups on the antibody following the periodate oxidation (Figure 3). We found that using NaIO_4_ at 10 mM at RT provided the highest conversion to aldehyde (about three aldehyde group/antibody molecules) of the antibody carbohydrates (Figure S2).

#### 3.1.2. Recognition Efficiency and Quality Assessment of Ox-1B4

As a further step, we evaluated whether oxidation affected the binding efficiency of the antibody to its antigen. The experiment, which was performed through a direct ELISA, showed that the Ox-1B4 and Un-1B4 at 1 μg/mL could similarly bind Cripto-1 at different concentrations (0–1.5–6–24 nM) (Figure S3), suggesting that oxidation did not alter the antigen recognition site.

Next, we evaluated the ability of the Ox-1B4 to be immobilized on a solid surface in an oriented configuration and the potentially improved ability to recognize the target antigen. The experiments were performed on a flat gold surface functionalized with SH-PEG-NH_2_ for the oriented immobilization and on SH-PEG-COOH to achieve a random immobilization. The Ox-1B4 was anchored on the SH-PEG-NH_2_ at 0–5–15–30 μg/mL, and the imine bond was stabilized via reduction. The 1B4 was randomly immobilized in concentrations ranging from 0 to 15 µg/mL following the amine coupling using SH-PEG-COOH. As shown in Figure S4, the oriented 1B4 showed an immobilization level that was almost double that of the random 1B4 at high concentrations. At the same time, it seemed that the random 1B4 had already reached a plateau at 5 μg/mL as opposed to the oriented one that saturated at 15 µg/mL. These data showed that the orientation of the antibody allowed the attachment of a greater quantity of the antibody, which in theory was even more predisposed to the recognition.

#### 3.1.3. SPR Assay Optimization on GC Device

**Figure 4:**
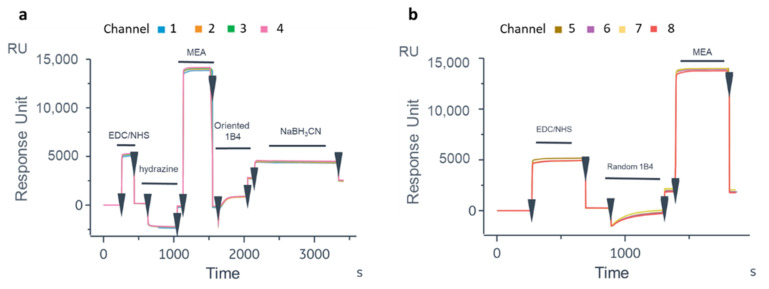
Sensorgrams obtained following immobilization of 1B4 by aldehyde coupling (oriented) on active FC2 (**a**) and following the random immobilization of 1B4 active FC2 (**b**) on the GC biosensor.

**Figure 5:**
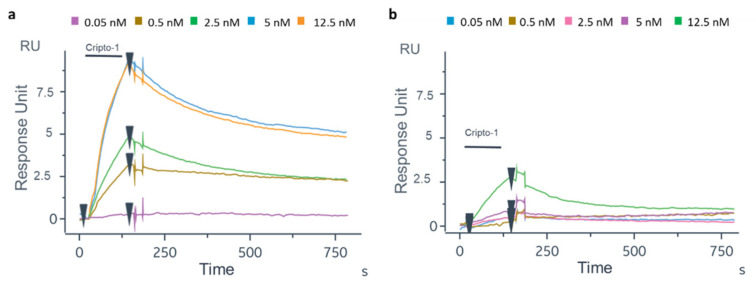
Sensorgrams related to the binding of Cripto-1 at different concentrations (0.05–0.5–2.5–5–12.5 nM) to oriented 1B4 (**a**) and the same antibody randomly immobilized (**b**).

To translate the protocol on the OFMTs’ gold tips, the first experiment was carried out on the GC biosensor to test the biofunctionalization protocol on a flat gold surface and verify the recognition efficiency of the oriented antibody. After the thiolation process, the Ox-1B4 was immobilized by following the aldehyde coupling method on the FC2 of the four channels. The 1B4 was instead randomly immobilized by the amine coupling procedure on the FC2 of the other four channels. As shown in Figure 4a, a higher level of 1B4 was immobilized in the oriented configuration (2489 ± 53 RU) compared to that achieved with the random immobilization (1886.75 ± 129.5 RU, Figure 4b). On the GC biosensor, an unrelated antibody in the oriented and random configurations was immobilized on FC1 (Figure S5) to account for the unspecific binding on the gold surface. After BSA blocking [62], solutions of Cripto-1 at different concentrations (0.05–0.5–2.5–5–12.5 nM) were injected onto the GC biosensor (Figure 5). The data highlighted that the oriented 1B4 was better able to recognize the antigen compared to the same antibody that was randomly immobilized (Table 1). A statistically significant recognition of the antigen by the oriented antibody was confirmed by the paired t-test analysis (*p* = 0.0087). The data showed that the randomly immobilized 1B4 failed to recognize the analyte at concentrations below 5 nM, while the oriented 1B4 detected Cripto-1 up to 0.05 nM. The sensorgrams also showed that at concentrations between 0.5 and 5 nM, the oriented antibody captured between 10 and 15 times more analyte (Table 1).

**Figure 6:**
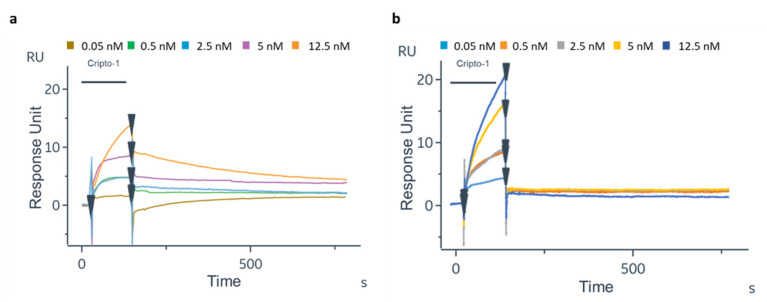
Sensorgrams related to the binding of Cripto-1 spiked at different concentrations (0.05–0.5–2.5–5–12.5 nM) in DMEM to oriented 1B4 (**a**) and to the same antibody randomly immobilized (**b**).

The experiments for Cripto-1 quantification also were performed in DMEM culture medium spiked with defined amounts of the protein. As shown in Figure 6a,b, Cripto-1 at 0.05–0.5–2.5–5–12.5 nM was well detected on both GC biosensors functionalized with oriented or random 1B4. However, the detection signal on the oriented 1B4 was much higher compared to the random one. Furthermore, a much faster dissociation of the antigen was observed in the case of the randomly immobilized antibody than in the case of the oriented antibody, suggesting that the antigen recognition under these conditions was probably more attributable to non-specific binding. The response signal on the sensor with the oriented 1B4 increased proportionally to the increasing Cripto-1 concentration, and the measured RU values exhibited a lower standard deviation. Finally, the randomly immobilized antibody was able to recognize Cripto-1 only at the highest concentration (12.5 nM), while the sensor with the oriented antibody successfully detected the analyte in the entire concentration range of 0.05 to 12.5 nM. The experiments in DMEM were carried out to verify only the performance of the GC biosensor in the detection of real samples with a complex matrix. DMEM is the most common cell culture medium and was therefore a good model for Cripto-1 quantification in a real setting [63].

### 3.2. Lab-on-Fiber Sensing Platforms

Once the optimal functionalization strategy was identified using the GC biosensor, this was transferred onto two different label free LoF platforms:

– A BLI-based platform exploiting a plastic optical fiber;
– An advanced LoF biosensor based on highly sensitive OFMTs.

#### 3.2.1. Cripto-1 Detection through BLI

**Figure 7:**
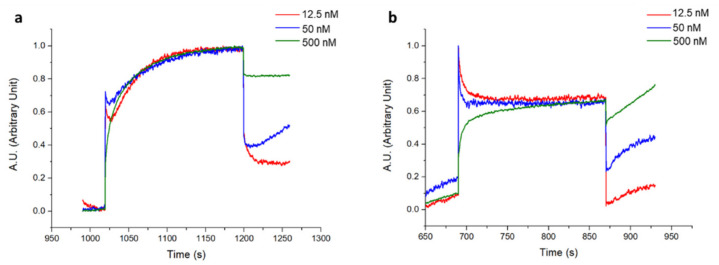
Normalized interferograms obtained for Cripto-1 detection at different concentrations (12.5–50–500 nM) by 1B4 immobilized in oriented (**a**) and random (**b**) configurations.

The Cripto-1 detection was performed using a commercially available label-free platform based on BLI and using the single-channel BLItz system. The antibody was immobilized on the AR2G biosensor using aldehyde and amine coupling as in the Biacore 8K experiments. It should be noted that the immobilization level obtained on the sensor chip for both antibodies was very similar, achieving a final shift of about 2 nm (Figure S6). After antibody immobilization, Cripto-1 was incubated at concentrations varying between 0 and 500 nM (0–12.5–50–500 nM). The corresponding normalized interferograms are reported in Figure 7, while the measured shifts are shown in Table 2. Although the protein could not be detected on this platform at the nM concentrations explored with the GC biosensor, the data confirmed that the oriented 1B4 had an almost tripled capability to detect the antigen at low concentrations (12.5 nM and 50 nM) compared to the random 1B4 (Table 2). A statistically significant recognition of the antigen by the oriented antibody was confirmed via the paired *t*-test analysis (*p* = 0.0189). Saturation was reached for both antibodies at a concentration of 500 nM; no protein was detected at concentrations below 12.5 nM. The experiments carried out using the plastic optical fiber of the BLItz, which had a much smaller surface area than the GC biosensor, confirmed that the antibody immobilized in an oriented way recognized Cripto-1 more effectively than the random one. However, the method lacked sensitivity, so the minimum concentration of the antigen (0.05 nM) that could instead be detected with the GC biosensor was not reached.

#### 3.2.2. Cripto-1 Detection through OFMT-Assisted Biosensors

To improve the LoF platform’s sensing performances, Cripto-1 detection was also conducted using the advanced OFMT-based platform, a potential POC device that combined the advantages of the lab-on-chip and lab-on-fiber paradigms.

**Figure 8:**
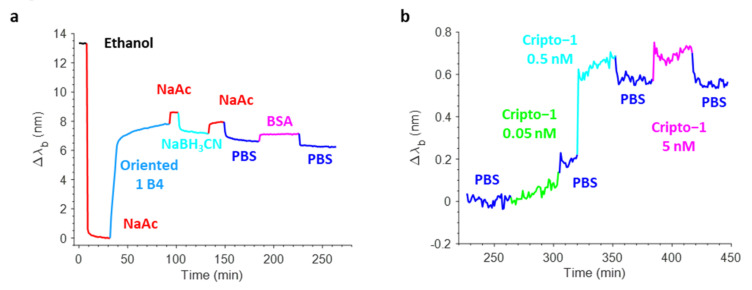
Real-time sensorgram showing the variations in the barycentric wavelength of the resonance of an OFMT during the biofunctionalization phase (**a**). Sensorgram related to the detection of Cripto-1 at increasing concentrations in PBS in the range 0.05–5 nM (**b**).

Figure 8a shows a typical real-time sensorgram obtained during the biofunctionalization of the OFMT transducer along with the variations in the barycentric wavelength pertaining to the resonant valley in the reflection spectrum (Δλ_b_) during the antibody immobilization (aldehyde coupling), the stabilization with NaBH_3_CN, and the blocking phase in BSA. Specifically, the incubation of the OFMT with 15 µg/mL Ox-1B4 in NaAc caused a strong variation in the resonance wavelength of about 8 nm due to the rapid and progressive change in the local refractive index induced by the binding of the antibody on the OFMT-activated surface. The successive NaAc wash led only to a small redshift in the Δλ_b_, thus affording a clear demonstration of the effective covalent immobilization of the oriented 1B4. Contrarily, the incubation with NaBH_3_CN led to a small decrease in the Δλ_b_, which may have been associated with the stabilization of the antibodies attached to the surface of the OFMTs. The passivation step that consisted of incubation with 3% *w*/*v* BSA in PBS resulted in a slight redshift as a consequence of the higher bulk refractive index of the BSA rather than the PBS. The shift measured before and after BSA passivation was slightly negative, suggesting that the antibody substantially saturated the available coupling sites.

Once biofunctionalized, the OFMT probe prototype was tested for Cripto-1 detection at different concentrations. Figure 8b shows the sensorgram obtained in real-time monitoring of the variations in the resonant wavelength of the OFMT probe during a preliminary experiment carried out using Cripto-1 at 0.05–0.5–5 nM in PBS. The OFMT biosensor showed an increase in Δλ_b_ after exposure to the Cripto-1 solution that was only minimally restored after washing with the buffer. It is important to point out that the detection of Cripto-1 by the OFMT was based on the formation of a bond between the bioreceptor and the target molecule on the activated surface of the biosensor. This bond had the effect of introducing a local modification of the refractive index, which resulted in a redshift of the resonant wavelength. The higher the analyte concentration, the greater the shift. At low concentrations, the biosensor response was initially very weak because the few bonded target molecules did not form a sufficiently homogeneous layer to determine a significant change in the local refractive index on the OFMT. As the number of bound target molecules increased, a faster growth of the sensor response was observed until it reached a saturation condition when there were no longer any free binding sites on the antibodies to anchor new target molecules. Referring to Figure 8b, the OFMT probe was able to detect Cripto-1 even at concentrations of 0.05 and 0.5 nM, with responses of 0.2 nm and 0.6 nm, respectively. In addition, exposure to the solution at 5 nM promoted no further signal increase; the probe response appeared to be substantially saturated at this concentration. This was attributed to the small sensitive surface of the OFMT, which featured a patterned area of ~14 × 14 μm^2^ centered inside the 50 × 50 μm^2^ gold square and positioned within the central part of the fiber end facet.

**Figure 9:**
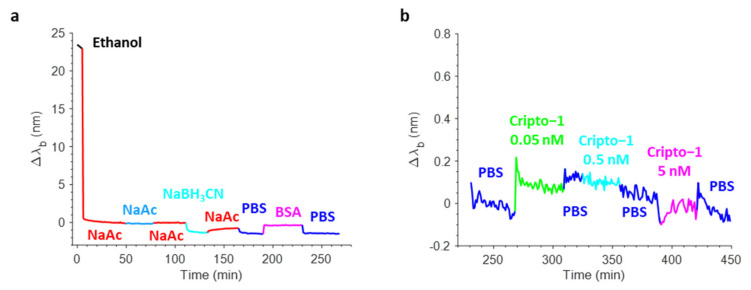
Real-time sensorgram showing the variations in the barycentric wavelength of the resonance of an OFMT during the biofunctionalization phase without the oriented 1B4 antibody (**a**). Probe immersion at increasing concentrations of Cripto-1 in PBS in the range of 0.05 and 0.5 nM (**b**).

The sensorgrams recorded on a non-biofunctionalized OFMT probe are shown in Figure 9. Here, the 1B4 was not immobilized, but the surface underwent a process similar to the previous one. The lack of the antibody during the immobilization step did not determine the increase in the sensor response. The response also was very weak during washes and during the reduction and BSA passivation steps (Figure 9a). Figure 9b shows the sensorgram recorded following exposure of the non-functionalized OFMT probe to solutions of Cripto-1 at 0.05, 0.5, and 5 nM. The sensorgram shows maximum variations in Δλ_b_ of 0.12 nm; i.e., much lower than the response achieved with the functionalized OFMT probe.

The results obtained using the OFMT functionalized with the oriented 1B4 revealed the huge potentiality of this novel LoF biosensing platform to detect Cripto-1 at very low concentrations, equalling those attained with the Biacore 8K benchtop reference (0.05 nM). These experiments also demonstrated the capability of the OFMT biosensor to outperform the results provided by the commercial BLItz system employing commercial optical fiber probes by a factor of 250.

To the best of our knowledge, this represents the first label-free system based on OFMTs that was adapted to the detection of Cripto-1. The integration with a microfluidic system and the use of polymeric matrices for the fiber tip biofunctionalization, which were able to significantly increase the amount of anchored antibody on the fiber tip surface by replacing the 2D area with an enhanced 3D volume, may have significantly improved the final detection capability. To sustain this conjecture, we also carried out mSPR experiments using the oriented 1B4 on a standard CM5 chip equipped with a carboxymethylated dextran, which created a 3D layer that allowed for more functional groups that were available for antibody binding. The obtained results demonstrated that a 3D polymer matrix coupled with a fine microfluidic system allowed for a higher level of antibody immobilization (Figure S7a), thus leading to a better molecular target recognition efficiency of about one order of magnitude higher (Figure S7b, Table S1). This approach provided extra degrees of freedom for further improvements in the overall performances as a result of the integration of a 3D dextran matrix on the active gold surface of the proposed platforms. At the same time, the reported comparison reinforced once again the significant impact of the proposed oriented antibody strategy on the achieved performances. This revolutionary strategy paves the way for an improvement in the performance of OFMTs, which could evolve through the integration of a polymer matrix and a suitable flow-management system.

## 4. Conclusions

To respond to the continuously growing need for innovative and ever-sensitive methods to detect biological targets, in this paper we investigated the possibility of improving the performances of two promising LoF biosensing platforms; i.e., a commercial biolayer interferometry (BLI)-based device, which was used as a benchmark, and an advanced homemade LoF biosensor that was based on highly sensitive OFMTs.

The improvement was achieved thanks to an oriented homemade antibody immobilization strategy that was obtained through hinge carbohydrate oxidation and provided a target for site-directed immobilization.

In particular, we assessed a pipeline for an effective translation of the optimized biofunctionalization protocol to the LoF platforms, which allowed us to minimize errors and avoid complex and time-consuming assays using the LoF platforms.

Specifically, we first tested and assessed the biofunctionalization strategy using a fast and well-established commercial mSPR system, the Biacore 8K. Next, once we identified the optimal functionalization protocol, this was transferred to the two label-free LoF platforms.

The validation of the oriented antibody and the best recognition of the target were performed for a relevant case study; i.e., Cripto-1, an emerging tumor biomarker for breast cancer with pathological levels in the blood in the range of 1–3 ng/mL [40] (0.05–0.15 nM).

Noticeably, the reported results demonstrated that an oriented antibody immobilization strategy was able to significantly improve Cripto-1 detection, enhancing the sensing capability by about 10-fold versus the random approach. More interestingly, the OFMT-based platform was able to reveal Cripto-1 at a concentration of 0.05 nM, exhibiting detection capabilities much higher (by a factor of 250) than those provided by the commercial LoF platform based on BLI and similar to the ones shown by the commercial and well-established mSPR Biacore 8K system.

We also demonstrated through mSPR experiments using the oriented immobilized 1B4 antibody on a chip equipped with a carboxymethylated dextran matrix that the use of a 3D dextran matrix for the biofunctionalization of an active gold-coated OFMT surface may provide additional degrees of freedom for a further improvement in its biosensing performances.

## Figures and Tables

**Table 1:** RU measurements of the binding of Cripto-1 to oriented 1B4 and to randomly immobilized 1B4 on GC biosensors. Each point is the mean of three different experiments.

Cripto-1 (nM)	Oriented 1B4	Random 1B4
	ΔRU	
0.05	0.75 ± 0.07	0.35 ± 0.07
0.5	3.90 ± 0.40	0.35 ± 0.35
2.5	5.05 ± 0.20	0.35 ± 0.07
5	7.35 ± 0.07	0.76 ± 0.15
12.5	7.50 ± 1.97	1.86 ± 0.60

**Table 2:** Normalized BLI shift values (nm) measured during Cripto-1 detection at different concentrations (12.5–50–500 nM) by oriented 1B4 and random 1B4. Each point is the mean of three different experiments.

Shift Ab (nm)		Cripto-1 (nM)	Shift Cripto-1 (nm)	
Oriented 1B4 30 μg/mL	Random 1B4 30 μg/mL		Oriented 1B4	Random 1B4
2.06 ± 0.45	2.36 ± 0.62	0	0.001 ± 0.0006	0.001 ± 0.00025
		12.5	0.33 ± 0.04	0.11 ± 0.08
		50	0.49 ± 0.08	0.20 ± 0.07
		500	0.84 ± 0.03	0.7 ± 0.17

## Data Availability

Not applicable.

## Supplementary Materials

The following supporting information can be downloaded at: https://www.mdpi.com/article/10.3390/bios12111040/s1, Figure S1: Optimization of the LyCH conjugation reaction with the antibody aldehydes. Untreated 1B4 was used as a negative control; Figure S2: Optimization of the periodate oxidation condition on 1B4 varying periodate concentration and reaction temperature; Figure S3: Dose-dependent recognition efficiency of Cripto-1 by Ox-1B4 and Un-1B4. Both anti-bodies were used at 1 μg/mL; Figure S4: Quality assessment of antibody: coupling of different concentrations (x-axes) of oriented 1B4 (a) and random 1B4 (b) on gold functionalized with specific thiol. Response Fluorescence Unit (RFU) divided by mm2 of the well on the y-axis; Figure S5: Sensorgrams were obtained following immobilization of an unrelated antibody by aldehyde coupling (oriented) (a) and of an unrelated antibody by amine coupling (random) (b) on Reference FC1 of GC biosensor; Figure S6: Immobilization of 1B4 by aldehyde coupling (oriented immobilization) (a), and by amine coupling to achieve random immobilization (b); Figure S7: Sensorgram related to the immobilization on CM5 chip of 1B4 by aldehyde coupling (a) and to the binding of Cripto-1 at different concentrations (0.05–0.5–2.5–5–12.5 nM) (b); Table S1: ∆RU measured for the binding of Cripto-1 to oriented 1B4 on CM5 chip.
