# Supplementary material for: Advanced Lab-on-Fiber Optrodes Assisted by Oriented Antibody Immobilization Strategy
Source: Biosensors (Basel). 2022 Nov 17;12(11):1040. doi: 10.3390/bios12111040 (PMC9688615; doi:10.3390/bios12111040)
Supplement: Supplementary file 1 [file biosensors-12-01040-s001.zip › biosensors-1941098-supplementary.pdf]

biosensors

MDPI

Supporting Information

Advanced Lab-on-Fiber Optrodes Assisted by Oriented Antibody Immobilization Strategy

Sarassunta Ucci [1], Sara Spaziani [2,3,*], Giuseppe Quero [2,3], Patrizio Vaiano [2], Maria Principe [2], Alberto Micco [3], Annamaria Sandomenico [1], Menotti Ruvo [1], Marco Consales [2,3] and Andrea Cusano [2,3]

[1] Institute of Biostructures and Bioimaging, National Research Council of Italy, via P. Castellino, 111, 80131 Napoli, Italy
[2] Optoelectronics Group, Engineering Department, University of Sannio, c.so Garibaldi 107, 82100 Benevento, Italy
[3] Centro Regionale Information Communication Technology (CeRICT Scrl), 82100 Benevento, Italy
* Correspondence: sara.spaziani@cerict.it

Content

1. **Antibody Labeling Efficiency** S-2
2. **Optimization Antibody Reduction** S-2
3. **Recognition Efficiency and Quality Assessment of Ox-1B4** S-2
4. **Immobilization of Unrelated Antibodies on GC Biosensor** S-3
5. **1B4 Antibody Immobilization for Cripto-1 Detection Through BLItz System** S-4
6. **1B4 Antibody Immobilization for Cripto-1 Detection Through CM5** S-4

References S-5

1. Antibody Labeling Efficiency

The effective number of aldehyde groups available for coupling was determined experimentally by LyCH labeling [61]. In Figure S1 it is possible to observe that the best conjugation between LyCH and Ox-1B4 occurred using a 1:250 ratio between the antibody and the dye.

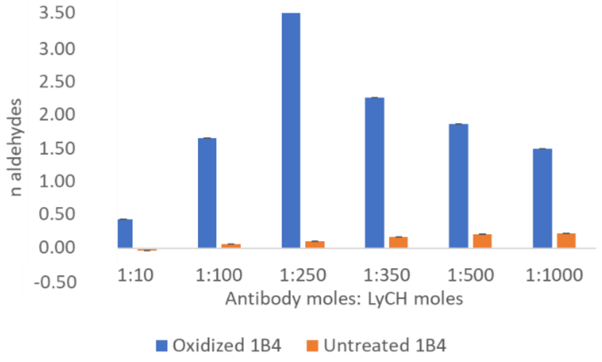

Figure S1. Optimization of the LyCH conjugation reaction with the antibody aldehydes. Untreated 1B4 was used as a negative control.

2. Optimization Antibody Reduction

We also optimized the reaction conditions to obtain as many aldehyde groups on the antibody as possible after the periodate oxidation. We found that using $NaIO_4$ at 10 mM at room temperature provided the highest conversion to aldehyde (approximately 3 antibody molecules/aldehyde group) of the antibody carbohydrates (Figure S2).

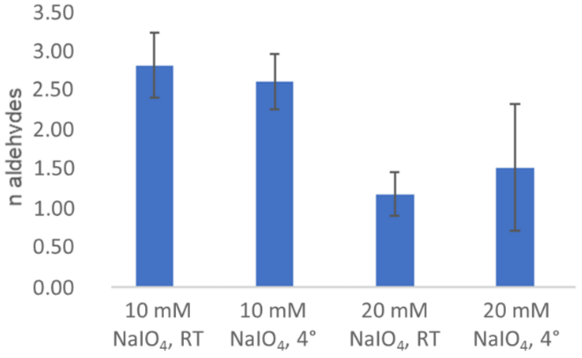

Figure S2. Optimization of the periodate oxidation condition on 1B4 varying periodate concentration and reaction temperature.

3. Recognition Efficiency and Quality Assessment of Ox-1B4

We evaluated whether oxidation affected the binding efficiency of the antibody to its antigen performing a direct ELISA (Figure S3).

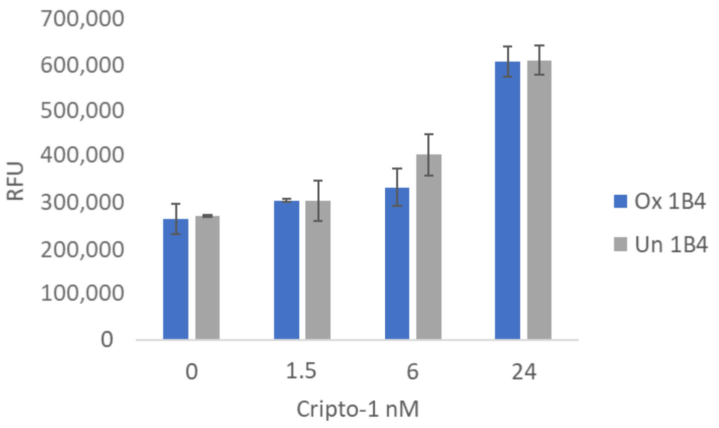

Figure S3. Dose-dependent recognition efficiency of Cripto-1 by Ox-1B4 and Un-1B4. Both antibodies were used at 1 µg/mL.

Successively, we evaluated the ability of Ox-1B4 to be immobilized on a solid surface in an oriented configuration. The experiments were performed on a flat gold surface functionalized with SH-PEG-NH_2 for the oriented immobilization and on SH-PEG-COOH to achieve random immobilization (Figure S4).

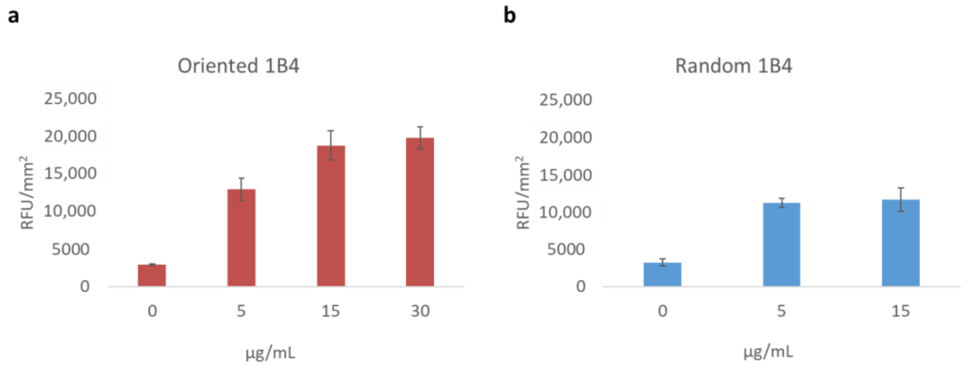

Figure S4. Quality assessment of antibody: coupling of different concentrations (x-axes) of oriented 1B4 (**a**) and random 1B4 (**b**) on gold functionalized with specific thiol. Response Fluorescence Unit (RFU) divided by mm2 of the well on the y-axis.

4. Immobilization of Unrelated Antibodies on GC Biosensor

After the thiolation process, Ox-1B4 was immobilized following the aldehyde coupling method on the FC2 of four channels. 1B4 was instead randomly immobilized by the amine coupling procedure on the FC2 of the other four channels. On the GC biosensor, an unrelated antibody in the oriented (1491 ± 29 RU) (Figure S5a) and random (622,975 ± 32,6 RU) (Figure S5b) configuration was immobilized on FC1 to account for the unspecific binding on the gold surface.

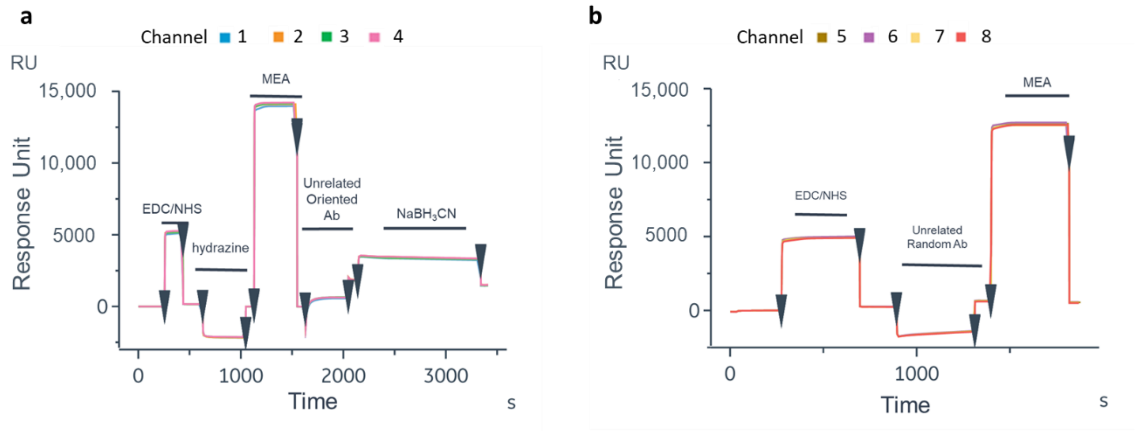

Figure S5. Sensorgrams were obtained following immobilization of an unrelated antibody by aldehyde coupling (oriented) (**a**) and of an unrelated antibody by amine coupling (random) (**b**) on Reference FC1 of GC biosensor.

5. 1B4 Antibody Immobilization for Cripto-1 Detection Through BLItz System

Cripto-1 detection was also performed by means of a second commercially available label-free platform based on BLI using the single-channel BLItz system. The antibody was immobilized onto the AR2G biosensor by aldehyde and amine coupling as in Biacore 8K experiments (Figure S6). It should be noted that the immobilization level obtained on the sensor chip of both antibodies was very similar achieving a final shift of about 2 nm.

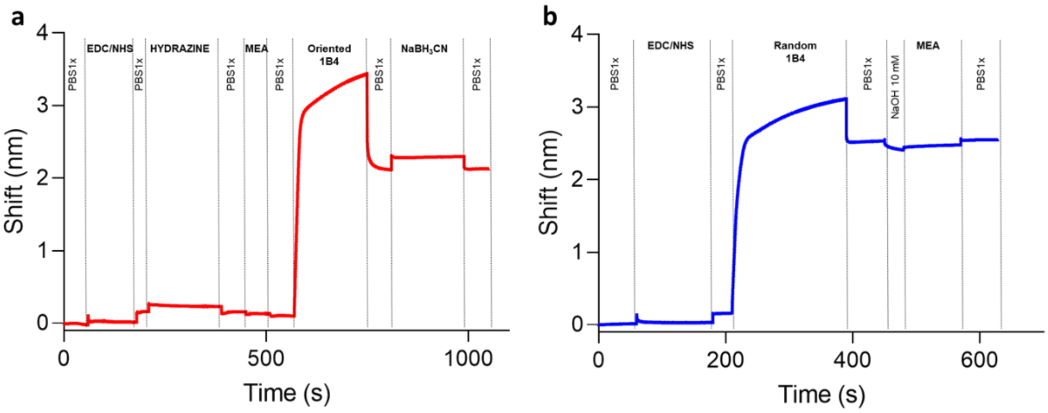

Figure S6. Immobilization of 1B4 by aldehyde coupling (oriented immobilization) (**a**), and by amine coupling to achieve random immobilization (**b**).

6. 1B4 Antibody Immobilization for Cripto-1 Detection Through CM5

A CM5 chip, a commercial chip functionalized with carboxymethylated dextran, was mounted in the Biacore 8K and primed with HBS-EP+. BIAnormalizing solution 70% was used to calibrate the detector. The coupling procedures for the oriented antibody were the same described for the GC biosensor. Ox-1B4 was immobilized on the Active Flow Cell 2 (FC2) (26810 RU Figure S7a) of one of the eight channels using the aldehyde coupling chemistry. Reference Flow Cell 1 (FC1) was treated in the same way but without antibody injection. The Multi Cycle Kinetics (MCK) option was adopted to evaluate the interaction between Cripto-1 and 1B4. MCK consists of parallel injection of analytes at increasing concentrations in separate cycles and regeneration of the sensor surface after each sample injection with 5 mM sodium hydroxide. Cripto-1 was analyzed at 0.05 – 0.5 – 2.5 – 5 – 12.5 nM (Figure S7b).

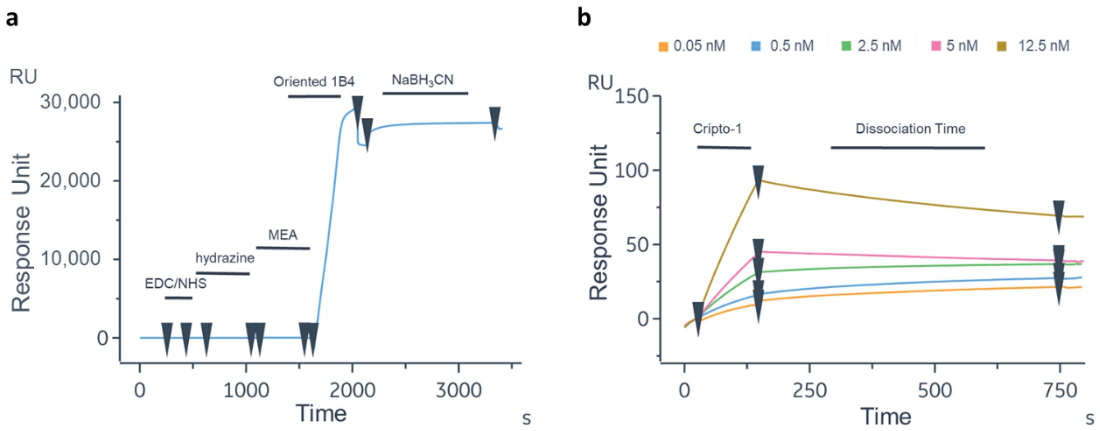

Figure S7. Sensorgram related to the immobilization on CM5 chip of 1B4 by aldehyde coupling (**a**) and to the binding of Cripto-1 at different concentrations (0.05 – 0.5 – 2.5 – 5 – 12.5 nM) (**b**).

Table S1. ΔRU measured for the binding of Cripto-1 to oriented 1B4 on CM5 chip.

Cripto-1 (nM)	Oriented 1B4 ΔRU
0.05	21.75 ± 0.49
0.5	26.65 ± 1.06
2.5	35.80 ± 1.41
5	38.35 ± 1.34
12.5	68.70 ± 1.13

References

61. Wolfe, C.A.C.; Hage, D.S. Studies on the Rate and Control of Antibody Oxidation by Periodate. Anal. Biochem. **1995**, 231, 123–130. https://doi.org/10.1006/abio.1995.1511.
